# *N*-(Benzothia­zol-2-yl)butyramide

**DOI:** 10.1107/S1600536808020667

**Published:** 2008-07-12

**Authors:** Sohail Saeed, Moazzam Hussain Bhatti, Peter G. Jones

**Affiliations:** a Department of Chemistry, Allama Iqbal Open University, Islamabad, Pakistan; b Institut für Anorganische und Analytische Chemie, Technische Universität Braunschweig, Postfach 3329, 38023 Braunschweig, Germany

## Abstract

The title compound, C_11_H_12_N_2_OS, was synthesized from 2-amino­benzothia­zole and butanoyl chloride in anhydrous acetone. In the crystal structure, mol­ecules are linked by N—H⋯N and C—H⋯O hydrogen bonds and by C—H⋯π inter­actions.

## Related literature

For related literature, see: Butt *et al.* (2005 ▶); Im & Jung (2000 ▶); Yang *et al.* (2002 ▶); Ataei *et al.* (2005 ▶).
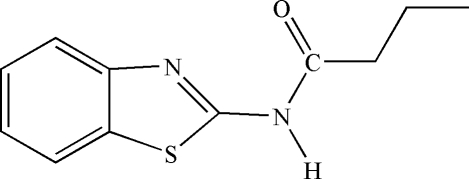


## Experimental

### 

#### Crystal data

C_11_H_12_N_2_OS
*M* *_r_* = 220.29
Triclinic, 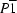

*a* = 5.2916 (4) Å
*b* = 7.4462 (8) Å
*c* = 13.565 (1) Å
α = 92.618 (7)°
β = 90.607 (6)°
γ = 107.185 (8)°
*V* = 509.92 (8) Å^3^
*Z* = 2
Mo *K*α radiation
μ = 0.29 mm^−1^
*T* = 100 (2) K
0.35 × 0.20 × 0.05 mm

#### Data collection

Oxford Diffraction Xcalibur S diffractometer
Absorption correction: multi-scan (*CrysAlis RED*; Oxford Diffraction, 2008 ▶) *T*_min_ = 0.977, *T*_max_ = 1.000 (expected range = 0.963–0.986)
8577 measured reflections
2728 independent reflections
2172 reflections with *I* > 2σ(*I*)
*R*_int_ = 0.035

#### Refinement

*R*[*F* ^2^ > 2σ(*F* ^2^)] = 0.035
*wR*(*F* ^2^) = 0.090
*S* = 0.99
2728 reflections
141 parameters
H atoms treated by a mixture of independent and constrained refinement
Δρ_max_ = 0.41 e Å^−3^
Δρ_min_ = −0.26 e Å^−3^

### 

Data collection: *CrysAlis CCD* (Oxford Diffraction, 2008 ▶); cell refinement: *CrysAlis RED* (Oxford Diffraction, 2008 ▶); data reduction: *CrysAlis RED*; program(s) used to solve structure: *SHELXS97* (Sheldrick, 2008 ▶); program(s) used to refine structure: *SHELXL97* (Sheldrick, 2008 ▶); molecular graphics: *XP* (Siemens, 1994 ▶); software used to prepare material for publication: *SHELXL97*.

## Supplementary Material

Crystal structure: contains datablocks I, global. DOI: 10.1107/S1600536808020667/im2075sup1.cif

Structure factors: contains datablocks I. DOI: 10.1107/S1600536808020667/im2075Isup2.hkl

Additional supplementary materials:  crystallographic information; 3D view; checkCIF report

## Figures and Tables

**Table 1:** Hydrogen-bond geometry (Å, °)

*D*—H⋯*A*	*D*—H	H⋯*A*	*D*⋯*A*	*D*—H⋯*A*
N1—H1⋯N2^i^	0.84 (2)	2.40 (2)	3.232 (2)	172 (1)
C10—H10⋯O^ii^	0.95	2.46	3.277 (2)	144
C2—H2*B*⋯*Cg*1^iii^	0.99	2.66	3.56	152
C3—H3*B*⋯*Cg*1^iv^	0.99	2.64	3.47	142

Symmetry codes: (i) 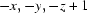; (ii) 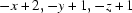; (iii) 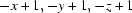; (iv) 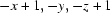. *Cg*1 is the centroid of the C6–C11 ring.

**Fig. 1.**
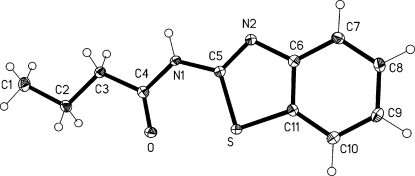
The molecular structure of the title compound. Ellipsoids represent 50% probability levels.

**Fig. 2.**
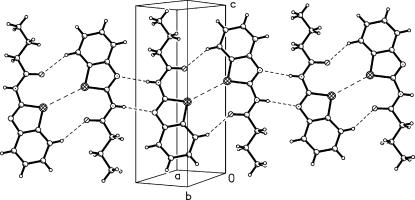
Packing diagram of I showing classical and "weak" H bonds and S···S contacts as thin dashed bonds. View direction is perpendicular to [102].

## supplementary crystallographic information

### Comment

High temperature polymers have received much attention due to the increasing demands for the replacement of ceramics and metals (Ataei *et al.*, 2005). However, in many cases, they are insoluble and do not melt below their decomposition temperature,which restricts their applications (Im & Jung, 2000). Thus, many studies have focused on obtaining aromatic polymers that are processable by conventional techniques (Yang *et al.*, 2002). The title compound, (I), is a precursor for an attempt to synthesize polyimides (Butt *et al.*, 2005), imidazole derivatives and transition metal complexes. The entire molecule (except H atoms) is planar within a mean deviation of 0.03 Å. Molecules are connected in ribbons parallel to [210] by classical hydrogen bonds N1—H1···N2 and additional weak hydrogen bonds C10—H10···O. S atoms of neighbouring molecules approach each other to 3.5267 (7) Å. Perpendicular to the ribbons are two C—H···π interactions (Table 1, Fig.2). The molecular structure of the title compound is depicted in Figure 1.

### Experimental

A mixture of butanoyl chloride (0.1 mol) and 2-aminobenzothiazole (0.1 mol) in anhydrous acetone (75 ml) was refluxed for 20 h. After cooling, the reaction mixture was poured in acidified cold water. The resulting dark brown solid was filtered and washed with cold acetone. Crystals of the title compound (I) suitable for X-Ray analysis were obtained after re-crystallization of the solid from ethanol (2.36 g, 79%). m.p.447 K.

### Refinement

The NH hydrogen was refined freely. Methyl H atoms were included on the basis of an idealized rigid group (C—H 0.98 Å, H—C—H 109.5°) allowed to rotate but not tip. Other hydrogen atoms were included using a riding model with C—H 0.95 (aromatic) or 0.99 (methylene) Å. U(H) values were fixed at 1.5*U*_iso_(C) of the parent C atom for methyl H, 1.2*U*_iso_(C) for other H.

### Crystal data

**Table:**

C_11_H_12_N_2_OS	*Z* = 2
*M**_r_* = 220.29	*F*_000_ = 232
Triclinic, *P*1	*D*_x_ = 1.435 Mg m^−^^3^
Hall symbol: -P 1	Mo *K*α radiation λ = 0.71073 Å
*a* = 5.2916 (4) Å	Cell parameters from 4460 reflections
*b* = 7.4462 (8) Å	θ = 2.9–30.7º
*c* = 13.565 (1) Å	µ = 0.29 mm^−^^1^
α = 92.618 (7)º	*T* = 100 (2) K
β = 90.607 (6)º	Plate, yellow
γ = 107.185 (8)º	0.35 × 0.20 × 0.05 mm
*V* = 509.92 (8) Å^3^	

### Data collection

**Table:**

Oxford Diffraction Xcalibur S diffractometer	2728 independent reflections
Radiation source: Enhance (Mo) X-ray Source	2172 reflections with *I* > 2σ(*I*)
Monochromator: graphite	*R*_int_ = 0.035
Detector resolution: 16.1057 pixels mm^-1^	θ_max_ = 30.0º
*T* = 100(2) K	θ_min_ = 2.9º
ω scans	*h* = −7→7
Absorption correction: multi-scan(CrysAlis RED; Oxford Diffraction, 2008)	*k* = −9→10
*T*_min_ = 0.977, *T*_max_ = 1.000	*l* = −19→18
8577 measured reflections	

### Refinement

**Table:**

Refinement on *F*^2^	Secondary atom site location: difference Fourier map
Least-squares matrix: full	Hydrogen site location: inferred from neighbouring sites
*R*[*F*^2^ > 2σ(*F*^2^)] = 0.035	H atoms treated by a mixture of independent and constrained refinement
*wR*(*F*^2^) = 0.091	*w* = 1/[σ^2^(*F*_o_^2^) + (0.0544*P*)^2^]
*S* = 0.99	(Δ/σ)_max_ = 0.001
2728 reflections	Δρ_max_ = 0.41 e Å^−^^3^
141 parameters	Δρ_min_ = −0.26 e Å^−^^3^
Primary atom site location: structure-invariant direct methods	Extinction correction: none

### Special details

**Table:**

Geometry. All e.s.d.'s (except the e.s.d. in the dihedral angle between two l.s. planes) are estimated using the full covariance matrix. The cell e.s.d.'s are taken into account individually in the estimation of e.s.d.'s in distances, angles and torsion angles; correlations between e.s.d.'s in cell parameters are only used when they are defined by crystal symmetry. An approximate (isotropic) treatment of cell e.s.d.'s is used for estimating e.s.d.'s involving l.s. planes.Non-bonded distance3.5267 (0.0007) S - S_$2 $2 - *x* + 2, -*y* + 1, -*z* + 1Least-squares planes (*x*,*y*,*z* in crystal coordinates) and deviations from them (* indicates atom used to define plane)- 2.9402 (0.0016) *x* + 6.9014 (0.0013) *y* + 2.6739 (0.0017) *z* = 1.5591 (0.0012)* -0.0482 (0.0011) N1 * -0.0353 (0.0010) N2 * 0.0176 (0.0010) O * -0.0420 (0.0006) S * 0.0121 (0.0012) C1 * 0.0438 (0.0012) C2 * 0.0205 (0.0013) C3 * 0.0008 (0.0013) C4 * -0.0386 (0.0013) C5 * -0.0125 (0.0012) C6 * 0.0334 (0.0011) C7 * 0.0608 (0.0012) C8 * 0.0306 (0.0012) C9 * -0.0160 (0.0011) C10 * -0.0269 (0.0012) C11Rms deviation of fitted atoms = 0.0332
Refinement. Refinement of *F*^2^ against ALL reflections. The weighted *R*-factor *wR* and goodness of fit *S* are based on *F*^2^, conventional *R*-factors *R* are based on *F*, with *F* set to zero for negative *F*^2^. The threshold expression of *F*^2^ > σ(*F*^2^) is used only for calculating *R*-factors(gt) *etc*. and is not relevant to the choice of reflections for refinement. *R*-factors based on *F*^2^ are statistically about twice as large as those based on *F*, and *R*- factors based on ALL data will be even larger.

### Fractional atomic coordinates and isotropic or equivalent isotropic displacement parameters (Å^2^)

**Table:**

	*x*	*y*	*z*	*U*_iso_*/*U*_eq_	
N1	0.3005 (2)	0.17657 (16)	0.43971 (8)	0.0115 (2)	
H1	0.142 (3)	0.110 (2)	0.4321 (11)	0.019 (4)*	
N2	0.2856 (2)	0.10743 (16)	0.60669 (8)	0.0110 (2)	
O	0.65683 (18)	0.36649 (14)	0.36601 (7)	0.0168 (2)	
S	0.74719 (6)	0.32370 (5)	0.55350 (2)	0.01216 (11)	
C1	0.2219 (3)	0.2887 (2)	0.08646 (10)	0.0186 (3)	
H1A	0.1669	0.1526	0.0710	0.028*	
H1B	0.3186	0.3540	0.0309	0.028*	
H1C	0.0653	0.3306	0.0981	0.028*	
C2	0.4007 (3)	0.3337 (2)	0.17869 (9)	0.0141 (3)	
H2A	0.5602	0.2931	0.1665	0.017*	
H2B	0.4589	0.4715	0.1934	0.017*	
C3	0.2581 (2)	0.2353 (2)	0.26720 (9)	0.0120 (3)	
H3A	0.1017	0.2794	0.2799	0.014*	
H3B	0.1935	0.0983	0.2506	0.014*	
C4	0.4267 (2)	0.26838 (19)	0.35982 (9)	0.0118 (3)	
C5	0.4162 (2)	0.19131 (19)	0.53257 (9)	0.0102 (3)	
C6	0.4520 (2)	0.14896 (19)	0.69100 (9)	0.0109 (3)	
C7	0.3830 (3)	0.0893 (2)	0.78621 (9)	0.0130 (3)	
H7	0.2093	0.0123	0.7986	0.016*	
C8	0.5711 (3)	0.1442 (2)	0.86165 (9)	0.0142 (3)	
H8	0.5244	0.1051	0.9264	0.017*	
C9	0.8288 (3)	0.2560 (2)	0.84506 (10)	0.0145 (3)	
H9	0.9547	0.2907	0.8983	0.017*	
C10	0.9017 (3)	0.3166 (2)	0.75156 (10)	0.0133 (3)	
H10	1.0764	0.3922	0.7395	0.016*	
C11	0.7103 (3)	0.26286 (19)	0.67562 (9)	0.0110 (3)	

### Atomic displacement parameters (Å^2^)

**Table:**

	*U*^11^	*U*^22^	*U*^33^	*U*^12^	*U*^13^	*U*^23^
N1	0.0082 (5)	0.0137 (6)	0.0107 (5)	0.0002 (5)	−0.0016 (4)	0.0019 (4)
N2	0.0099 (5)	0.0118 (6)	0.0109 (5)	0.0026 (4)	0.0002 (4)	0.0006 (4)
O	0.0122 (4)	0.0210 (6)	0.0135 (5)	−0.0013 (4)	0.0001 (4)	0.0041 (4)
S	0.00957 (16)	0.0143 (2)	0.01083 (16)	0.00062 (12)	0.00010 (11)	0.00174 (12)
C1	0.0215 (7)	0.0210 (8)	0.0123 (6)	0.0046 (6)	−0.0013 (5)	0.0027 (6)
C2	0.0137 (6)	0.0167 (8)	0.0120 (6)	0.0043 (6)	0.0009 (5)	0.0029 (5)
C3	0.0106 (6)	0.0132 (7)	0.0118 (6)	0.0030 (5)	−0.0010 (5)	0.0010 (5)
C4	0.0130 (6)	0.0107 (7)	0.0122 (6)	0.0041 (5)	0.0009 (5)	0.0006 (5)
C5	0.0104 (6)	0.0090 (7)	0.0116 (6)	0.0034 (5)	0.0000 (4)	0.0006 (5)
C6	0.0115 (6)	0.0102 (7)	0.0120 (6)	0.0048 (5)	−0.0003 (5)	−0.0007 (5)
C7	0.0116 (6)	0.0137 (7)	0.0139 (6)	0.0036 (5)	0.0018 (5)	0.0025 (5)
C8	0.0164 (6)	0.0166 (8)	0.0110 (6)	0.0067 (6)	0.0008 (5)	0.0024 (5)
C9	0.0142 (6)	0.0155 (8)	0.0142 (6)	0.0055 (5)	−0.0040 (5)	−0.0007 (5)
C10	0.0123 (6)	0.0125 (7)	0.0148 (6)	0.0036 (5)	−0.0012 (5)	−0.0004 (5)
C11	0.0120 (6)	0.0111 (7)	0.0108 (6)	0.0048 (5)	0.0009 (4)	0.0013 (5)

### Geometric parameters (Å, °)

**Table:**

N1—C4	1.3788 (16)	C9—C10	1.3858 (18)
N1—C5	1.3803 (16)	C10—C11	1.3956 (18)
N2—C5	1.3022 (16)	N1—H1	0.841 (17)
N2—C6	1.4015 (16)	C1—H1A	0.9800
O—C4	1.2209 (16)	C1—H1B	0.9800
S—C11	1.7351 (13)	C1—H1C	0.9800
S—C5	1.7501 (13)	C2—H2A	0.9900
C1—C2	1.5238 (18)	C2—H2B	0.9900
C2—C3	1.5236 (17)	C3—H3A	0.9900
C3—C4	1.5011 (17)	C3—H3B	0.9900
C6—C7	1.4019 (17)	C7—H7	0.9500
C6—C11	1.4027 (18)	C8—H8	0.9500
C7—C8	1.3802 (18)	C9—H9	0.9500
C8—C9	1.3988 (19)	C10—H10	0.9500
			
C4—N1—C5	123.92 (11)	C2—C1—H1B	109.5
C5—N2—C6	108.90 (10)	H1A—C1—H1B	109.5
C11—S—C5	87.62 (6)	C2—C1—H1C	109.5
C3—C2—C1	111.29 (11)	H1A—C1—H1C	109.5
C4—C3—C2	113.98 (11)	H1B—C1—H1C	109.5
O—C4—N1	121.46 (12)	C3—C2—H2A	109.4
O—C4—C3	124.23 (11)	C1—C2—H2A	109.4
N1—C4—C3	114.30 (11)	C3—C2—H2B	109.4
N2—C5—N1	121.66 (11)	C1—C2—H2B	109.4
N2—C5—S	118.00 (9)	H2A—C2—H2B	108.0
N1—C5—S	120.34 (9)	C4—C3—H3A	108.8
N2—C6—C7	126.34 (12)	C2—C3—H3A	108.8
N2—C6—C11	114.83 (11)	C4—C3—H3B	108.8
C7—C6—C11	118.83 (12)	C2—C3—H3B	108.8
C8—C7—C6	119.00 (12)	H3A—C3—H3B	107.7
C7—C8—C9	121.58 (12)	C8—C7—H7	120.5
C10—C9—C8	120.45 (12)	C6—C7—H7	120.5
C9—C10—C11	117.88 (12)	C7—C8—H8	119.2
C10—C11—C6	122.25 (12)	C9—C8—H8	119.2
C10—C11—S	127.12 (10)	C10—C9—H9	119.8
C6—C11—S	110.63 (9)	C8—C9—H9	119.8
C4—N1—H1	118.4 (11)	C9—C10—H10	121.1
C5—N1—H1	117.7 (11)	C11—C10—H10	121.1
C2—C1—H1A	109.5		
			
C1—C2—C3—C4	177.95 (12)	N2—C6—C7—C8	−179.52 (13)
C5—N1—C4—O	2.2 (2)	C11—C6—C7—C8	−0.1 (2)
C5—N1—C4—C3	−178.25 (12)	C6—C7—C8—C9	−0.6 (2)
C2—C3—C4—O	0.8 (2)	C7—C8—C9—C10	0.6 (2)
C2—C3—C4—N1	−178.73 (11)	C8—C9—C10—C11	0.2 (2)
C6—N2—C5—N1	−179.44 (12)	C9—C10—C11—C6	−1.0 (2)
C6—N2—C5—S	0.99 (15)	C9—C10—C11—S	178.35 (10)
C4—N1—C5—N2	177.35 (12)	N2—C6—C11—C10	−179.60 (12)
C4—N1—C5—S	−3.09 (18)	C7—C6—C11—C10	0.9 (2)
C11—S—C5—N2	−0.39 (11)	N2—C6—C11—S	0.99 (15)
C11—S—C5—N1	−179.97 (12)	C7—C6—C11—S	−178.49 (10)
C5—N2—C6—C7	178.19 (13)	C5—S—C11—C10	−179.73 (14)
C5—N2—C6—C11	−1.25 (17)	C5—S—C11—C6	−0.35 (10)

### Hydrogen-bond geometry (Å, °)

**Table:**

*D*—H···*A*	*D*—H	H···*A*	*D*···*A*	*D*—H···*A*
N1—H1···N2^i^	0.84 (2)	2.40 (2)	3.232 (2)	172 (1)
C10—H10···O^ii^	0.95	2.46	3.277 (2)	144
C2—H2B···Cent(C6–C11)^iii^	0.99	2.66	3.56	152
C3—H3B···Cent(C6–C11)^iv^	0.99	2.64	3.47	142

Symmetry codes: (i) −*x*, −*y*, −*z*+1; (ii) −*x*+2, −*y*+1, −*z*+1; (iii) −*x*+1, −*y*+1, −*z*+1; (iv) −*x*+1, −*y*, −*z*+1.
